# Structural basis of increased binding affinities of spikes from SARS-CoV-2 Omicron variants to rabbit and hare ACE2s reveals the expanding host tendency

**DOI:** 10.1128/mbio.02988-23

**Published:** 2023-12-19

**Authors:** Kaiyuan Shi, Linjie Li, Chunliang Luo, Zepeng Xu, Baihan Huang, Sufang Ma, Kefang Liu, Guanghui Yu, George F. Gao

**Affiliations:** 1 Hubei Provincial Key Laboratory for Protection and Application of Special Plants in Wuling Area of China, College of Life Sciences, South-Central Minzu University, Wuhan, China; 2 CAS Key Laboratory of Pathogen Microbiology and Immunology, Institute of Microbiology, Chinese Academy of Sciences, Beijing, China; 3 College of Veterinary Medicine, Shanxi Agricultural University, Jinzhong, China; 4 Faculty of Health Sciences, University of Macau, Macau, China; Fondazione Biotecnopolo di Siena, Siena, Italy

**Keywords:** SARS-CoV-2, SARS-CoV, RBD, rabbit, hare, ACE2, cryo-EM structure, spike (S) proteins

## Abstract

The potential host range of severe acute respiratory syndrome coronavirus 2 (SARS-CoV-2) has been expanding alongside its evolution during the pandemic, with rabbits and hares being considered important potential hosts, supported by a report of rabbit sero-prevalence in nature. We measured the binding affinities of rabbit and hare angiotensin-converting enzyme 2 (ACE2) with receptor-binding domains (RBDs) from SARS-CoV, SARS-CoV-2, and its variants and found that rabbit and hare ACE2s had broad variant tropism, with significantly enhanced affinities to Omicron BA.4/5 and its subsequent-emerged sub-variants (>10 fold). The structures of rabbit ACE2 complexed with either SARS-CoV-2 prototype (PT) or Omicron BA.4/5 spike (S) proteins were determined, thereby unveiling the importance of rabbit ACE2 Q34 in RBD-interaction and elucidating the molecular basis of the enhanced binding with Omicron BA.4/5 RBD. These results address the highly enhanced risk of rabbits infecting SARS-CoV-2 Omicron sub-variants and the importance of constant surveillance.

**IMPORTANCE:**

The severe acute respiratory syndrome coronavirus 2 (SARS-CoV-2) pandemic has swept the globe and caused immense health and economic damage. SARS-CoV-2 has demonstrated a broad host range, indicating a high risk of interspecies transmission and adaptive mutation. Therefore, constant monitoring for potential hosts is of immense importance. In this study, we found that Omicron BA.4/5 and subsequent-emerged sub-variants exhibited enhanced binding to both rabbit and hare angiotensin-converting enzyme 2 (ACE2), and we elucidated the structural mechanism of their recognition. From the structure, we found that Q34, a unique residue of rabbit ACE2 compared to other ACE2 orthologs, plays an important role in ACE2 recognition. These results address the probability of rabbits/hares being potential hosts of SARS-CoV-2 and broaden our knowledge regarding the molecular mechanism of SARS-CoV-2 interspecies transmission.

## INTRODUCTION

Since the outbreak of Coronavirus Disease 2019 (COVID-19), the causative agent of severe acute respiratory syndrome coronavirus 2 (SARS-CoV-2) has been evolving, and new variants keep emerging across the world. In late 2021, the fifth variant of concern (VOC), Omicron, emerged and has been further evolving into multiple sub-variants with distinct characteristics, among which the host range alteration is of particular concern, as interspecies transmission could provide opportunities for unpredictable mutations and even give birth to new variants (1–5). As of June 27, 2023, 26 species, covering pets, livestock, and captive and wild animals, have been reported to be naturally infected by SARS-CoV-2 (https://www.woah.org/en/document/sars-cov-2-in-animals-situation-report-21/), whereas some other species have been reported to be susceptible to experimental infections (2, 6, 7). In particular, Omicron sub-variants have been sequenced from cat (BA.1, BA.1.1, BA.4, and BA.5), deer (BA.1.1), mink (BA.2, BA.5, and XBB.1.5), dog (XBB.1.5), hamster (BA.2.12 and BA.4), gorilla (BA.5), northern greater galago (XBB.1.5), and squirrel monkey (BA.2), indicating their susceptibility (https://gisaid.org/).

Receptor recognition and binding are the prerequisites of virus invasion and a key criterion for susceptibility assessment in computational and biochemical assays. SARS-CoV-2 uses the receptor binding domain (RBD) of spike (S) protein to recognize its host receptor angiotensin-converting enzyme 2 (ACE2) and mediate cell entry (8–10). Multiple studies have evaluated the receptor binding spectra of SARS-CoV-2 and revealed a broad potential host range, including bats, domestic animals, marine mammals, and other wild animals (1, 11–14). Thus far, plural structures of SARS-CoV-2 RBD binding to ACE2 orthologs from potential hosts have been solved, including bat, pangolin, mouse, civet, mink, horse, marine mammals, and companion animals (1, 12, 15–22). However, many important potential hosts remain to be investigated further.

Contradicting results have been reported regarding the susceptibility of rabbits to SARS-CoV-2. It has been reported that rabbit ACE2 binds strongly to both SARS-CoV and SARS-CoV-2 RBDs and efficiently mediates pseudovirus entry (12, 14). It is also reported that SARS-CoV-2 can replicate in renal cells from rabbits, and experimental infection proved rabbits susceptible to the SARS-CoV-2 prototype (PT) (BetaCoV/Munich/BavPat1/2020) and can support *in vivo* replication (23, 24). Another recent study reported SARS-CoV-2-binding antibodies in rabbit serum samples, suggesting a natural infection in domestic rabbits (25). However, another report suggests the insusceptibility of rabbits to SARS-CoV-2, where no detectable infectious viruses were shed in the rabbit inoculated with SARS-CoV-2 PT (2019-nCoV/USA-WA1/2020) (26). These contradictory results indicate that while the SARS-CoV-2 PT may infect rabbits, it does so with limited infectivity. Also, the hare is a close relative of rabbits and inhabits wild habitats (27), presenting a risk of “wild” community transmission. Considering that the Omicron variant demonstrated an altered potential host range (1), whether Omicron and its sub-variants have enhanced infectivity/transmissibility among rabbits and hares needs further investigation.

Herein, we assessed the binding affinities of ACE2 orthologs of rabbit and hare with SARS-CoV, SARS-CoV-2 PT, and important SARS-CoV-2 variants. We found that rabbit and hare ACE2s showed significantly strengthened binding with RBDs of Omicron BA.4/5 (Omicron BA.4 and BA.5 are combined as they share an identical RBD sequence) and subsequent sub-variants BF.7, BQ.1, BQ.1.1, XBB, and XBB.1.5, indicating a highly enhanced risk of rabbit infection with corresponding sub-variants. To elucidate the molecular basis of rabbit ACE2 binding to SARS-CoV, SARS-CoV-2 PT and Omicron BA.4/5 S proteins, we solved the cryo-EM structures of rabbit ACE2 in complex with S proteins of SARS-CoV, SARS-CoV-2 PT, and SARS-CoV-2 Omicron BA.4/5. Q34, a unique residue of rabbit ACE2 compared to other ACE2 orthologs, plays an important role in ACE2 recognition. These results address the increased risk of rabbits and hares infected with recently emerged SARS-CoV-2 Omicron sub-variants, BF.7, BQ.1, BQ.1.1, XBB, and XBB.1.5, and broaden the knowledge on the structural mechanism of SARS-CoV-2 interspecies transmission.

## RESULTS

### Rabbit ACE2 shows significantly enhanced affinity with RBDs of omicron BA.4/5 and subsequent sub-variants

**FIG 1:**
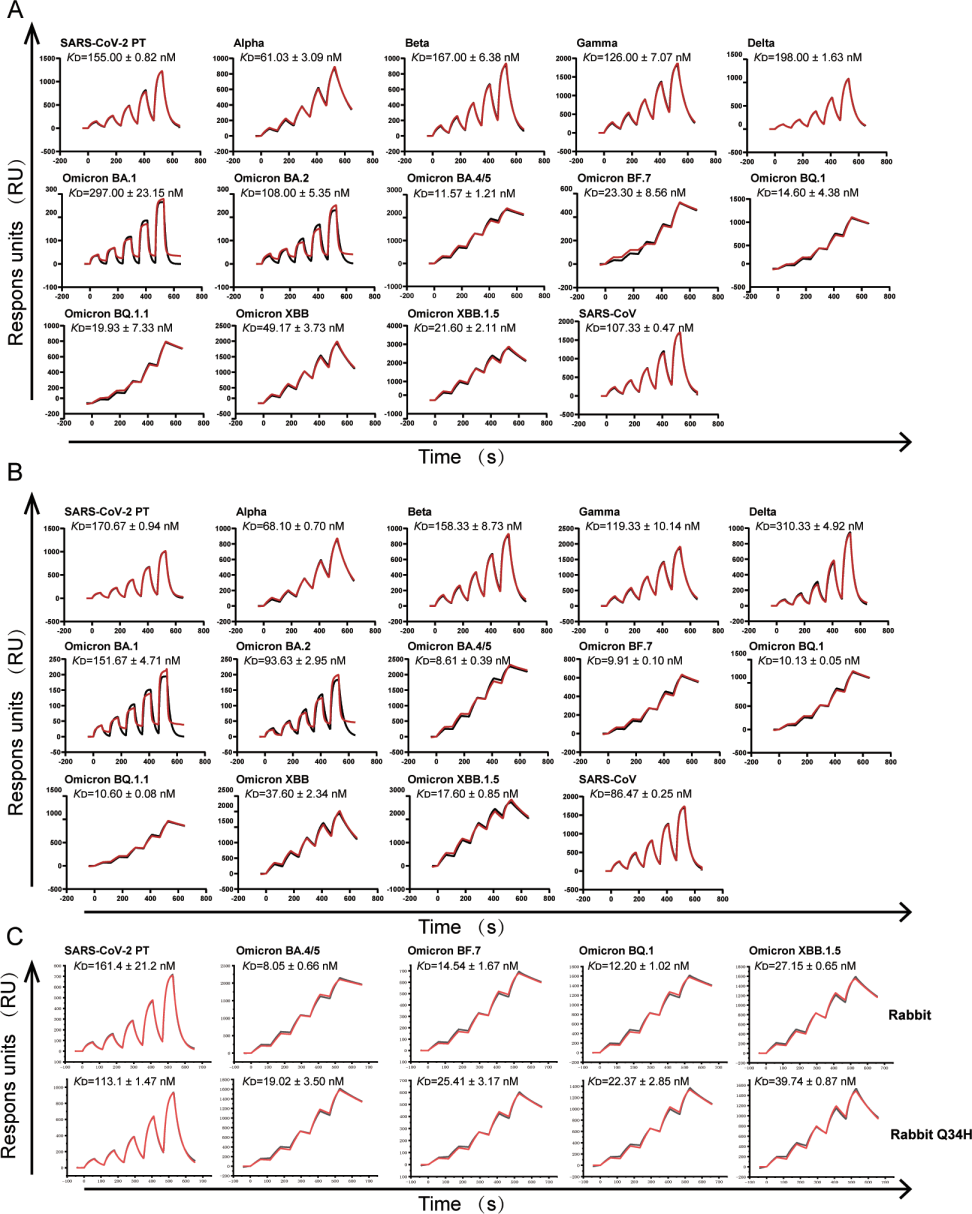
Rabbit and hare ACE2s broadly bind to RBDs of SARS-CoV, SARS-CoV-2, and SARS-CoV-2 variants. (**A and B**) The SPR assay of rabbit (**A**) and hare (**B**) ACE2s binding to RBDs of SARS-CoV, SARS-CoV-2 PT, and SARS-CoV-2 variants. (**C**) Comparison of rabbit ACE2 (upper) and rabbit ACE2 Q34H (lower) binding to SARS-CoV-2 PT and variants. Dissociation constants (*K*_D_) were calculated from three independent experiments and presented as mean ± standard deviation. The actual and fitted curves were colored in black and red, respectively.

The full-length amino acid identity of rabbit ACE2 and human ACE2 is 85.14%. Hare ACE2, on the other hand, contains 12 distinct residues from rabbit ACE2, none of which are located on the RBD binding interface based on human ACE2-RBD complex structures (Fig. S1). Among the residues binding with PT RBD (9), three substitutions are observed, namely, Q24L, D30E, and Q34H. The effect on receptor binding of the former two substitutions has been elucidated in our previous study (17), while Q34H has not been investigated yet. To evaluate the binding capacity of rabbit and hare ACE2s to RBDs of SARS-CoV, SARS-CoV-2, and its variants, we used surface plasmon resonance (SPR) to measure the binding affinities. The rabbit ACE2 receptor exhibits no significant difference in its binding affinity to the PT RBD when compared to its affinities for the first four SARS-CoV-2 variants of concern (Alpha, Beta, Gamma, and Delta), as well as for the Omicron variants BA.1 and BA.2 (*K*_D_ values from 61.0 nM to 297.0 nM). Meanwhile, Omicron BA.4/5 and subsequent Omicron sub-variants, BF.7, BQ.1, BQ.1.1, XBB, and XBB.1.5, showed significantly strengthened binding to rabbit ACE2s compared to PT (*K*_D_ values from 11.6 to 49.2 nM) (Fig. 1A). Hare ACE2 showed a similar pattern to rabbit ACE2. RBDs of SARS-CoV-2 PT and its variants before Omicron BA.2 bound to hare ACE2 with binding affinities ranging from 68.1 nM to 310.3 nM. Omicron BA.4/5 and RBDs of subsequent Omicron sub-variants bound to hare ACE2 with binding affinities from 8.6 nM to 37.6 nM (Fig. 1B), indicating again the enhanced binding. The SARS-CoV RBD bound to both rabbit and hare ACE2s with similar binding affinity in comparison with the SARS-CoV-2 PT RBD (Fig. 1A and B).

To assess the role of the unique amino acid Q34 of rabbit/hare ACE2, we further chose PT, BA.4/5, BF.7, BQ.1, and XBB.1.5 RBDs and measured their binding to wild-type rabbit ACE2, as well as to rabbit ACE2 mutants carrying Q34H substitution (Fig. 1C). PT RBD binds to rabbit ACE2 and rabbit ACE2 Q34H with comparable affinities, whereas rabbit ACE2 Q34H demonstrated decreased affinities to various extents when compared to the other four RBDs (1.46–2.36 fold).

### Overall architecture of the rabbit ACE2 in complex with S proteins of SARS-CoV-2 PT and omicron BA.4/5

**FIG 2:**
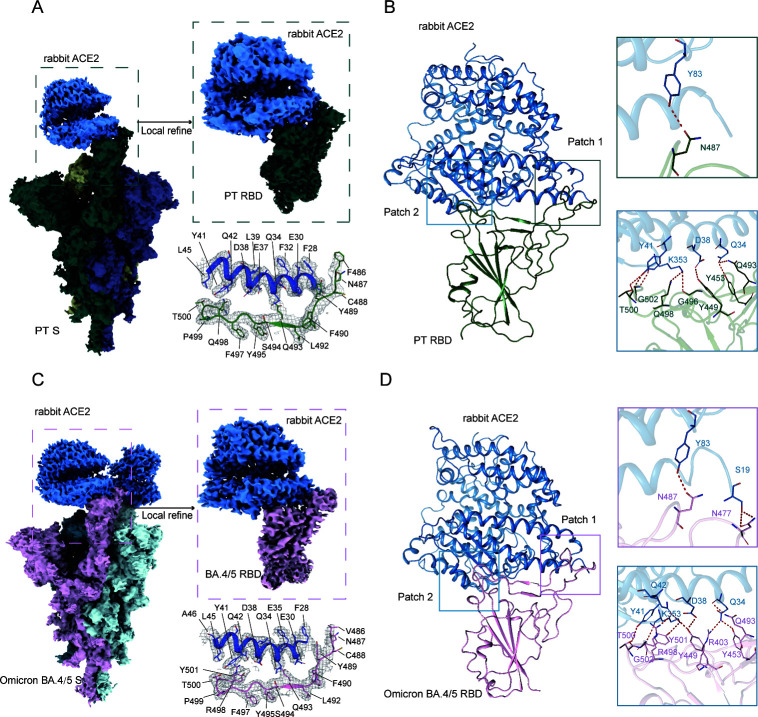
Structures of SARS-CoV-2 PT and omicron BA.4/5 S proteins in complex with rabbit ACE2. Cryo-EM density maps of the PT (**A**) and BA.4/5 (**C**) S proteins complexed with rabbit ACE2. The local refinement was performed at the binding interface of the RBD and rabbit ACE2. The density maps on the binding interface are shown as mesh. The overall structures of PT RBD/rabbit ACE2 (**B**) and Omicron BA.4/5 RBD/rabbit ACE2 (**D**) are shown as cartoons. The residues that participate in the H-bond networks of Patch 1 and Patch 2 are shown as sticks. The PT RBD and BA.4/5 RBD are colored in green and pink, respectively, and rabbit ACE2 in blue.

To elucidate the structural basis in rabbit ACE2 interacting with SARS-CoV-2 PT and the molecular mechanism of its enhanced binding affinities with Omicron BA.4/5 RBD, we solved the cryo-EM structures of rabbit ACE2 in complex with PT S and Omicron BA.4/5 S (Table S1). The structures were solved at 2.83 Å and 3.08 Å, respectively (Fig. 2A and C; Fig. S2 and S3). The PT S in the complex adopted a one-RBD-up conformation. Notably, although the same molar ratio of S and ACE2 (1: 3) were mixed during the sample preparation, the Omicron BA.4/5 S adopted a two-RBD-up conformation and bound with two ACE2s, indicating a more open conformation. The local refinement of the RBD/ACE2 interface of PT S/rabbit ACE2 and Omicron BA.4/5 S/rabbit ACE2 complexes harvested 2.75 Å and 3.14 Å molecular models, respectively. The density of interacting residues can be clearly observed (Fig. 2A and C).

The architecture of rabbit ACE2 binding to PT and Omicron BA.4/5 RBDs resembles that of PT RBD/human ACE2, with interacting residues on RBD divided into two patches (Fig. 2B and D). In Patch 1 of PT RBD/rabbit ACE2, Y83 of rabbit ACE2 forms a H-bond with N487 of the PT RBD, whereas in Patch 2, Q34, D38, Y41, and K353 form a H-bond network with Y449, Y453, Q493, G496, Q498, T500, and G502 (Fig. 2B). Omicron BA.4/5 RBD forms more H-bonds with rabbit ACE2 than that of PT RBD (12 vs. 9), which may attribute to the enhanced binding affinity (Table 1). In Patch 1, two additional H-bonds between S19 of rabbit ACE2 and N477 of Omicron BA.4/5 RBD were observed, whereas in Patch 2, Y449 of Omicron BA.4/5 RBD forms two H-bonds with D38 and Q42 of ACE2, and Y501 forms an H-bond with K353 (Fig. 2D).

**TABLE 1:** Comparison of the PT RBD, Omicron BA.4/5 RBD, and SARS-CoV RBD binding to rabbit ACE2^*a*^

Rabbit ACE2	PT RBD	Ba.4/5 RBD	SARS-CoV RBD
S19		N477 (2, 4)	D463 (2)
E23			P462 (7)
L24	G476 (1), N487 (3)	A475 (4), G476 (4), N487 (4)	P462 (2)
T27	F456 (6), Y473 (1), Y489 (4)	F456 (1), Y489 (7)	L443 (1), F460 (1), Y475 (4)
F28	Y489 (4)	Y489 (7)	Y475 (11)
E30	K417 (4), L455 (1), F456 (2)	L455 (1), F456 (1)	
K31	F456 (3), Y489 (5)	Q493 (5)	Y442 (5), Y475 (1)
Q34	Y453 (1, 5), L455 (1), Q493 (1, 22)	R403 (1, 8), Y453 (1, 6), Q493 (1, 11)	Y442 (8), L478 (1), N479 (1, 16)
E35	Q493 (1)	Q493 (2)	
E37	Y505 (1)		
D38	Y449 (1, 8), G496 (1)	Y449 (1, 5)	Y436 (1, 11)
Y41	Q498 (8), T500 (2, 6), N501 (5)	R498 (9), T500 (1, 7), Y501 (15)	Y484 (7), T486 (1, 5), T487 (5)
Q42	Y449 (1) ,Q498 (5)	Y449 (1, 1), R498 (1, 11)	Y436 (1, 5), Y484 (3)
L45		R498 (1)	S432 (1), Y484 (1), T486 (2)
L79		V486 (1)	L472 (1)
Y83	N487 (1, 3)	N487 (1, 4), Y489 (1)	N473 (1, 5), Y475 (1)
E329			R426 (1)
N330	T500 (4)	T500 (1)	T486 (1)
K353	G496 (1, 5), Q498 (1, 3), N501 (9), G502 (1, 3), Y505 (21)	G496 (1), Y501 (1, 20), G502 (1, 4), H505 (20)	Y481 (1, 3), G482 (1, 4), T487 (3), G488 (1, 3), Y491 (16)
G354	N501 (2), G502 (7), Y505 (1)	G502 (6)	G488 (6), Y491 (4)
D355	T500 (8)	T500 (4)	T486 (9), T487 (3), G488 (4)
R357	T500 (4)		T486 (3)
R393	Y505 (1)		Y491 (1)
TOTAL	169, **9**	176, **12**	167, **8**

^*a*^ The numbers in parentheses of the PT RBD, Omicron BA.4/5 RBD, and SARS-CoV RBD residues represent the number of Van der Waals' force (vdw) contacts between the indicated ACE2 residues with the RBDs. The underlined numbers indicate the number of potential H-bonds or salt bridges between the pairs of residues. The vdw contacts were analyzed at a cut-off of 4.5 Å. H-bonds or salt bridges were calculated using PDBePISA with a cut-off of 3.5 Å.

### Rabbit ACE2 induces RBD erection in SARS-CoV S/rabbit ACE2 complex

**FIG 3:**
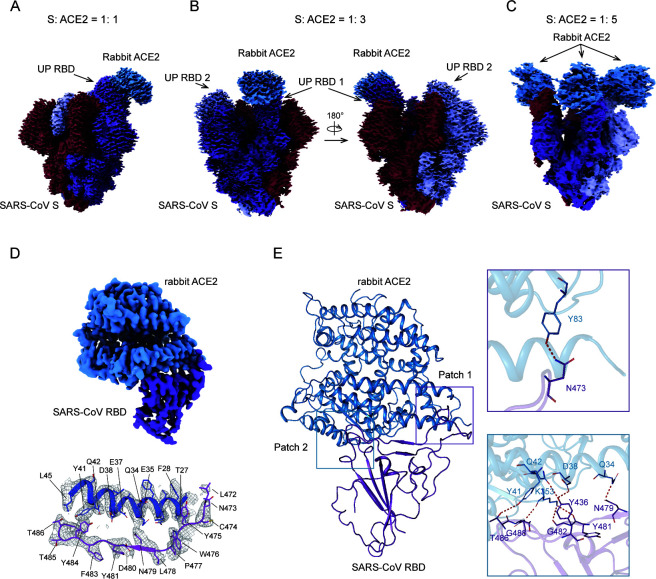
Rabbit ACE2-induced RBD erection in SARS-CoV S/rabbit ACE2 complex. (**A-C**) Cryo-EM density maps of SARS-CoV S/rabbit ACE2 complexes. The molar ratio of mixed S protein and ACE2 during sample preparation is labeled above the complexes. Three protomers of SARS-CoV S are colored in purple, red, and pale purple, respectively, and rabbit ACE2 in blue. (**D**) Cryo-EM density map of the SARS-CoV RBD/rabbit ACE2 complex and its binding interface. The main chains of interacting residues are presented as cartoons and side chains as sticks. The density of residues is presented as mesh. (**E**) Molecular model of SARS-CoV RBD/rabbit ACE2 complex and H-bond network. Residues involved in the H-bond network are shown as sticks.

To unveil the structural mechanism of SARS-CoV S recognizing rabbit ACE2, we solved the cryo-EM structure of SARS-CoV S in complex with rabbit ACE2 (Fig. S4). Interestingly, during the sample preparation, we found that the molar ratio of S and ACE2 proteins correlates with the RBD conformation of S protein (Fig. S4). When S: ACE2 = 1: 1, only one RBD is observed to adopt the up state (Fig. 3A). However, when S: ACE2 = 1: 3, although two RBDs are in the up state, only one of them is bound to rabbit ACE2, indicating a transition state where the ACE2-binding induces the erection of the adjacent RBD while the second ACE2 has not yet bound (Fig. 3B). When the composition of rabbit ACE2 was further increased to S: ACE2 = 1:5, all the RBDs were erected (Fig. 3C).

As the resolution of SARS-CoV S/rabbit ACE2 could not meet the required resolution for interface analysis, we further solved the cryo-EM structure of SARS-CoV RBD/rabbit ACE2 at 3.25 Å, allowing the interacting residues to be clearly observed (Fig. 3D, Fig. S5). The SARS-CoV RBD also binds to rabbit ACE2 in a classical pattern. The H-bond network is centered in Patch 2, except for a H-bond between Y83 and N473 in Patch 1. The ACE2 residues involved in H-bond interaction largely resemble those in the PT S/rabbit ACE2 complex. However, Q42 forms a H-bond with Y436 of SARS-CoV RBD, but it is not involved in the H-bond interaction with PT RBD (Fig. 3E). The other H-bond network involves Q34, D38, Y41, and K353 of rabbit ACE2 and N479, Y481, G482, T486, and G488 of SARS-CoV RBD.

### Distinct binding sites between human and rabbit ACE2

**FIG 4:**
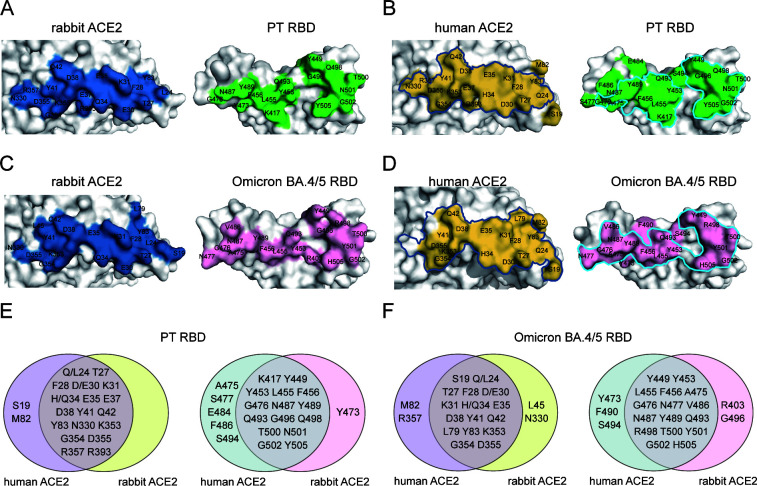
Comparison of the binding interface among PT and omicron BA.4/5 RBDs with human ACE2 or rabbit ACE2. (**A-D**) Binding interfaces of PT RBD/rabbit ACE2 (**A**), PT RBD/human ACE2 (**B**), Omicron BA.4/5 RBD/rabbit ACE2 (**C**), and Omicron BA.4/5 RBD/human ACE2 (**D**). Outline of the binding interface between rabbit ACE2 and designated RBDs is colored in blue and cyan, respectively. (**E and F**) Venn diagrams of key residues on the binding interface. PT RBD (**E**) and Omicron BA.4/5 RBD (**F**) residues involved in the interaction with human ACE2 and rabbit ACE2 are presented.

We compared the interface residues of human and rabbit ACE2 binding to PT and Omicron BA.4/5 RBD using the reported cryo-EM structures of PT RBD/human ACE2 (PDB: 7SXY) and Omicron BA.4/5 RBD/human ACE2 (PDB: 8H06) (28, 29). When binding to PT RBD, the S19 and M82 of human ACE2 participate in the interaction, whereas their rabbit ACE2 counterparts do not (Fig. 4A and B). More residues of PT RBD are involved in the recognition of human ACE2 than rabbit ACE2, where A475, S477, E484, F486, and S494 only interact with human ACE2 but not with rabbit ACE2 (Fig. 4E). When binding to Omicron BA.4/5 RBD, the RBD interfaces are largely overlapped, with Y473, F490, and S494 exclusively binding to human ACE2, and R403 and G496 exclusively binding to rabbit ACE2 (Fig. 4B and D). As for the ACE2 residues, M82 and R357 of human ACE2 take part in the RBD binding while their rabbit ACE2 counterparts do not, whereas L45 and N330 of rabbit ACE2 exclusively bind to the RBD with their human ACE2 counterparts not involved (Fig. 4C and F).

### Structural basis of enhanced binding of omicron BA.4/5 RBD to rabbit ACE2

**FIG 5:**
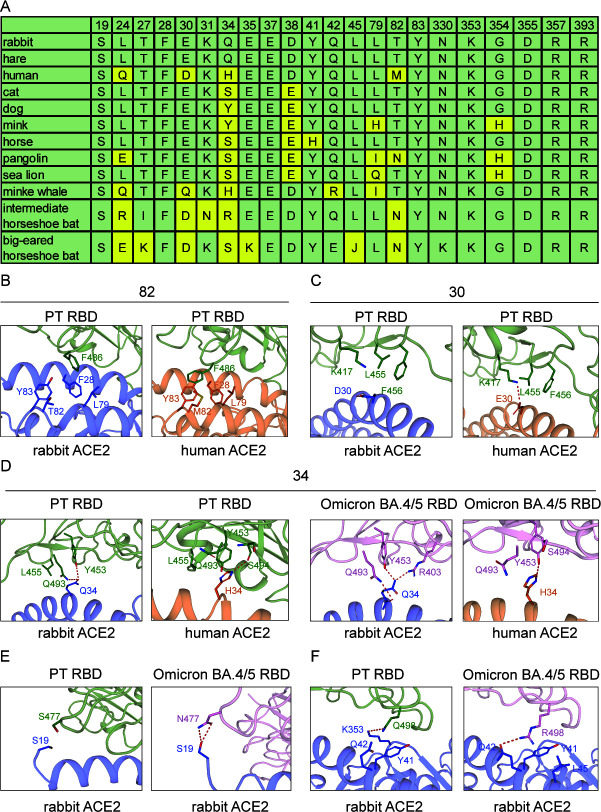
The role of mutated residues of variant RBDs in receptor binding. (**A**) Crucial ACE2 residues interacting with the PT RBD are listed. The highlighted residues indicate the distinctive residues among ACE2 orthologs. (**B-D**) Structural comparison of designated key ACE2 residues (labeled above) affecting RBD binding. The key residues are shown as sticks. The PT and Omicron BA.4/5 RBDs are colored in green and pink, respectively. The human ACE2 and rabbit ACE2 are in orange and blue, respectively. (**E and F**) Structural comparison of S477N (**E**) and Q498R (**F**) substitutions affects rabbit ACE2 binding.

To verify the structural role of the distinct binding sites between human and rabbit ACE2 and to elucidate the molecular basis of enhanced affinity between rabbit/hare ACE2s and Omicron BA.4/5 RBD, we further analyzed the structural details of key residues involved in the receptor binding. Consistent with the comparable binding affinities to SARS-CoV-2 RBDs, hare/rabbit ACE2s share identical RBD-interacting residues (Fig. 5A). For the difference between human ACE2 and rabbit ACE2, there are four distinct sites, namely, Q (human)/L (rabbit) 24, D/E30, H/Q34, and M/T82 (Fig. 5A; Table 1). Notably, Q34 is unique to rabbit ACE2 among all the species whose ACE2 complexed with SARS-CoV-2 RBD structures have been solved (Fig. 5A). Among the four distinct residues, Q/L24 and M/T82 were not involved in H-bond interactions (Table 1). However, M82 of human ACE2 participates in a hydrophobic patch composed of F28, L79, M82, Y83 of ACE2, and F486 of PT RBD. Yet, in rabbit ACE2, the hydrophilic T82 sabotages the hydrophobic patch, resulting in F486 being shifted away (Fig. 5B). The longer side chain of the human E30 residue forms a salt bridge with K417, whereas the D30 of rabbit ACE2 forms no H-bond with other residues (Fig. 5C). Q34, as a unique residue of rabbit ACE2, plays an important role in RBD recognition, forming two H-bonds with Q493 and Y453 of PT RBD, respectively. The H34 of human ACE2, on the other hand, forms only one H-bond with S494 of PT RBD (Fig. 5D).

As for the enhanced binding affinity of rabbit ACE2 with Omicron BA.4/5, Q493, N477, and R498 are shown to exert an essential effect. In the Omicron BA.4/5 RBD/rabbit ACE2 complex, R403 of the RBD directs toward Q34 of rabbit ACE2 and forms a H-bond in addition to the H-bonds with Q493 and Y453 (Fig. 5D). Besides, the S477N substitution of Omicron BA.4/5 RBD brings with it two additional H-bonds with S19 of rabbit ACE2, strengthening the receptor binding (Fig. 5E). The R498 of Omicron BA.4/5 RBD, on the other hand, forms a H-bond with Q42 instead of K353, and on top of that forms a π-cation interaction with Y41 (Fig. 5F).

## DISCUSSION

Since the identification of SARS-CoV-2, its potential host range has attracted great attention (2, 11, 30). The host range assessment based on the receptor binding capacity revealed a broad potential host range and guided animal host surveillance (1, 2, 11–13). For example, the susceptibility of deer and marine mammals has been predicted in computational and biochemical assays (11, 19), whose natural infection was observed later (31, 32). Notably, the follow-up investigation demonstrated that along with the evolution of SARS-CoV-2, its host range has been expanding (1). Thus, it is important to constantly monitor the potential host range and issue timely warnings on high-risk potential hosts. This study found that RBDs of Omicron BA.4/5 and subsequent sub-variants demonstrated significantly enhanced affinities with both rabbit and hare ACE2s, indicating a higher risk of SARS-CoV-2 infection. Thus, the surveillance of community spread among rabbits or hares is of great importance. Interestingly, a recent report demonstrated sero-prevalence of SARS-CoV-2 in rabbits, in which samples were collected from November 2020 to June 2021, a time slot largely coinciding with the outbreak of Omicron (25). These results suggest that rabbits are more at risk from Omicron sub-variants circulation and that the host tendency of SARS-CoV-2 is undergoing alteration. This addresses the importance of continuous evaluation of SARS-CoV-2 potential host range and constant surveillance for potential hosts.

The structures of SARS-CoV S complexed with rabbit ACE2 revealed a correlation between the proportion of ACE2 in sample preparation and S protein conformation, indicating an ACE2-induced RBD erection pattern. This phenomenon revealed that the conformation of RBD results from multiple factors acting in concert, and the observed number of erected RBDs in cryo-EM structures cannot be simply linked to the infectivity of the virus.

Rabbit ACE2 carries a unique Q34 residue, which plays an important role in increased affinity to Omicron BA.4/5. Q34 of rabbit ACE2 mainly interacts with Q493, which was substituted to R in the Omicron BA.1 variant and later underwent a reverse substitution event from the Omicron BA.4/5 sub-variant onwards. In our previous work, the R493Q reversion proved to increase binding affinities to ACE2 orthologs from humans and several animals, including rabbits (29). The fact that R493Q reversion was fixed in sub-variants emerging after Omicron BA.4/5 also indicates the evolutionary preference to Q493. Considering that Q493R is essential in immune evasion mutation and that the RBD mutations are the balance between receptor binding and immune evasion, can we courageously assume that after the long dominance of immune evasion in RBD evolution, receptor binding has again gained more weight and exerts a more significant effect? We believe that this assumption is worthwhile for further investigation.

Multiple other ACE2 residues have been found to affect SARS-CoV-2 recognition. Previously, we solved the crystal structure of SARS-CoV-2-RBD in complex with bat ACE2 from *Rhinolophus macrotis* (RmACE2), which contains Y41 and E42 at the corresponding sites, and found that ACE2 containing H41 and E42 could not bind to SARS-CoV-2-RBD, but the introduction of Q42 (H41-Q42) restored its RBD binding capacity. Moreover, studies on the structures of equine ACE2 complexed with SARS-CoV-2-RBD revealed that residue at site 41 (either H or Y) of ACE2 affects the binding to SARS-CoV-2-RBD and largely determines the preference for the residues at site 501 of SARS-CoV-2-RBD, where the H41-containing equine ACE2 showed weaker interactions with the N501Y mutant (17, 18). In addition, K31 and K353 have been reported as hotspots providing a substantial amount of energy for receptor binding, with K353A mutation perhaps abolishing the binding capacity of pangolin ACE2 to SARS-CoV-2-RBD (21, 33, 34). Structural analysis of SARS-CoV-2 RBD binding to ACE2 from intermediate horseshoe bats (*Rhinolophus affinis*) indicates that the E35K substitution, which alters the surface electric charge of ACE2 from negative to positive, plays an enhancing role in RBD recognition (16). These results may provide theoretical assistance with susceptibility evaluation.

In summary, we found that both rabbit and hare ACE2s broadly bound to RBDs from SARS-CoV, SARS-CoV-2, and its variants, and demonstrated the molecular mechanism of significantly enhanced affinities to Omicron BA.4/5 and subsequent sub-variants. These results addressed that Omicron BA.4/5 and subsequent sub-variants enhanced the possibility of rabbit and hare becoming their potential natural hosts, and broadened our knowledge regarding the structural mechanism of SARS-CoV-2 interspecies recognition.

## MATERIALS AND METHODS

### Cells

HEK293F suspension-cultured cells (Gibco, Cat# 11625–019) were cultured in SMM 293-TII expression medium (Sino Biological, Cat# M293TII) at 37°C.

### Gene cloning

For protein expression, 12 VOC RBDs (residues 319–541, GISAIDs Alpha: EPI_ISL_683466, Beta: EPI_ISL_678615, Gamma: EPI_ISL_833172, Delta: EPI_ISL_2020954, Omicron BA.1: EPI_ISL_6640916, Omicron BA.2: EPI_ISL_9845731, BA.4/5: EPI_ISL_12029894) were cloned into pCAGGS vector and BF.7 (BA.4/5 mutation R346T), BQ.1.1 (EPI_ISL_16098101), BQ.1 (BQ.1.1 mutation T346R), XBB (EPI_ISL_16096636), XBB.1.5 (XBB mutation S486P) into pCDNA3.1 vector. SARS-CoV RBD (residues 306–527, YP_009825051.1), and SARS-CoV-2 PT RBD (residues 319–541, GISAID: EPI_ISL_402119) were also cloned into the pCAGGS vector. SARS-CoV S-6P, SARS-CoV-2 PT S-6P, Omicron BA.4/5 S-6P-GSAS (residues 1–1237, 6P: R685S, F817P, A892P, A899P, A942P, K986P, V987P, GSAS: residue 681–684 PRRA mutated to GSAS) were cloned into pCAGGS vector. For SPR assays, the coding sequence of rabbit ACE2 (residues 1–615, Uniprot: G1TEF4) and HareACE2 (residues 1–615, GCA_009760805.1) were cloned into pCAGGS. For structure determination, the coding sequence of rabbit ACE2 (1-615) was transformed into pET21a vectors.

### Protein expression and purification

The proteins were expressed in HEK293F cells. Cell culture supernatants were collected after 5 days of transfection. Cell culture supernatants were collected, filtered through 0.22 µm filters, and purified by His-Trap HP (GE Healthcare) and SuperdexTM 200 Increase 10/300 Gl (GE Healthcare) size exclusion chromatography. The purified proteins were stored in a protein buffer (20 mM Tris-HCl, 150 mM NaCl, pH 8.0).

For the rabbit ACE2 used in structure determination, the plasmids were transformed into *E. coli* BL21 (DE3) cells and overexpressed as inclusion bodies under 1 mM IPTG induction. The inclusion bodies were then dissolved by dissolution buffer [6 M Gua-HCl, 10% vol/vol glycerol, 50 mM Tris-HCl, 100 mM NaCl, 10 mM ethylenediaminetetraacetic acid (EDTA), pH 8.0] and refolded. Briefly, 5 mL of the solution (30 mg/mL) was added drop by drop to 2.5 L refolding buffer [100 mM Tris-HCl, 400 mM L-Arg-HCl, 2 mM EDTA, 5 mM glutathione (GSH), and 0.5 mM oxidized glutathione (GSSG), pH 8.0]. After gently stirring for 8 h, we concentrated and exchanged proteins into a buffer containing 20 mM Tris-HCl (pH 8.0) and 150 mM NaCl and purified using a SuperdexTM 200 Increase 10/300 Gl column (GE Healthcare).

### SPR analysis

SARS-CoV RBD, SARS-CoV-2 PT RBD, VOC RBDs, rabbit ACE2, and hareACE2 were transferred to phosphate-buffered saline with Tween 20 buffer (1.8 mM KH_2_PO_4_, 10 mM Na_2_HPO_4_ (pH 7.4), 137 mM NaCl, 2.7 mM KCl, and 0.05% (vol/vol) Tween 20). RBD proteins were immobilized on a CM5 sensor (GE Healthcare). ACE2s were doubly diluted into five phases of concentration and then flowed through the CM5 chip using a single-cycle mode with the BIAcore 8K control system (GE Healthcare).

### Cryo-EM sample preparation and data acquisition

Before preparation of cryo-EM samples, PT S and Omicron BA.4/5 S were, respectively, incubated with *Escherichia coli*-expressed rabbit ACE2 at a molar ratio of 1:3 for 1 h on ice, and the concentration was adjusted to 3–5 mg/mL. After 15 mA/20 s glow discharge (PELCO easiGlow), the R1.2/1.3 copper 300-mesh grid (Quantifoil) was placed in the Vitrobot Mark IV (ThermoFisher Scientific). The grid was added with a 5 µL sample and waited for 10 s, then blotted for 6 s with the force of −4 and plunged into liquid ethane. For SARS-CoV S, the molar ratios of S and *E. coli*-expressed rabbit ACE2 are 1:1, 1:3, and 1:5, respectively, with other conditions unchanged. For SARS-CoV RBD, it was incubated with 293F-expressed rabbit ACE2 in a molar ratio of 2:1 on ice for 1 h, and the concentration was adjusted to 0.1 mg/mL. Without glow discharge, the R1.2/1.3 gold 300-mesh grid covered by graphene (Quantifoil) was added with a 5 µL sample, after waiting for 10 s, it was blotted for 2 s with the force of −1, and plunged into liquid ethane. All samples were prepared at 4°C and 100% humidity.

The grids were loaded into a 300 kV Titan Krios transmission electron microscope equipped with Gatan K3 direct electron detector (ThermoFisher Scientific). The S/ACE2 samples were exposed with a total dose of 50 e^-^/Å^2^ and the pixel size was 0.88 Å, while the RBD/ACE2 sample was exposed with a total dose of 60 e^-^/Å^2^ and the pixel size is 0.69 Å. Each movie collected by EPU software (ThermoFisher Scientific) contained 32 frames in counting mode, and the defocus range was −1.0 to −2.0 µm.

### Image processing

All the movie stacks were loaded into cryoSPARC v.3.3.1 (35) and then processed through MotionCor2 (36), the contrast transfer function estimation, blob picking and particle extraction, 2D classification, *ab initio* reconstruction, and 3D refinements. By the estimation of the gold-standard Fourier shell correlation cut-off value of 0.143, the resolutions were as follows: 2.83 Å for PT S/rabbit ACE2 global map, 2.75 Å for PT S/rabbit ACE2 local map, 3.08 Å for Omicron BA.4/5 S/rabbit ACE2 global map, 3.14 Å for Omicron BA.4/5 S/rabbit ACE2 local map, and 3.25 Å for SARS-CoV RBD /rabbit ACE2 map.

### Model building and structure refinement

For the model building of the S/ACE2 complexes, the SARS-CoV-2 S trimer with one hACE2 (PDB code 7DF4) was used as the initial model and fitted into the cryo-EM maps using UCSF Chimera X (37). Mutation and manual adjustment were carried out with CCP4-COOT v.0.9.3 (38). 10 rounds of the real-space refinement in Phenix-1.20.1 (39) were performed for each model. Molprobity (40) was used to validate geometry and estimate the quality of structures. Figures were generated by Chimera X (37) and PyMol v.2.0 (http://www.pymol.org).

### Statistics and reproducibility

*K*_D_ values for the binding study-SPR experiment were obtained using BIAcore Insight software (GE Healthcare), using a 1:1 binding model. The values shown are the mean ± SD of three replicates. All experiments were performed in biologically independent replicates.

## Data Availability

The atomic coordinates for the PT RBD/rabbit ACE2 complex, the Omicron BA.4/5 RBD/rabbit ACE2 complex, and the SARS-CoV RBD/rabbit ACE2 complex are deposited in the Protein Data Bank (https://www.rcsb.org/) with accession numbers of 8WOX, 8WOY, and 8WOZ, respectively. The corresponding electron microscopy maps of PT RBD/rabbit ACE2 complex, the Omicron BA.4/5 RBD/rabbit ACE2 complex, the SARS-CoV RBD/rabbit ACE2 complex, the PT S/rabbit ACE2, Omicron BA.4/5 S/rabbit ACE2, SARS-CoV S/rabbit ACE2 (1 RBD-up), SARS-CoV S/rabbit ACE2 (2 RBD-up), and SARS-CoV S/rabbit ACE2 (3 RBD-up) are deposited in the Electron Microscopy Data Bank with accession numbers EMD-37701, EMD-37702, EMD-37703, EMD-37704, EMD-37706, EMD-38137, EMD-38144, and EMD-38152, respectively.

## AFTER EPUB

[This article was published on 19 December 2023 with errors in the equal-contribution statement, Fig. 2, the Data Availability statement, and References. The errors were corrected in the current version, posted on 23 January 2024.]

## SUPPLEMENTAL MATERIAL

Supplemental material mbio.02988-23-s0001.docx Fig. F1-S5; Table S1.

ASM does not own the copyrights to Supplemental Material that may be linked to, or accessed through, an article. The authors have granted ASM a non-exclusive, world-wide license to publish the Supplemental Material files. Please contact the corresponding author directly for reuse.
