# Supplementary material for: Structural basis of increased binding affinities of spikes from SARS-CoV-2 Omicron variants to rabbit and hare ACE2s reveals the expanding host tendency
Source: mBio. 2023 Dec 19;15(2):e02988-23. doi: 10.1128/mbio.02988-23 (PMC10870819; doi:10.1128/mbio.02988-23)
Supplement: Supplemental material — Fig. F1-S5; Table S1. [file mbio.02988-23-s0001.docx]

**Supplementary Information**

**Structural basis of increased binding affinities of spikes from SARS-CoV-2 Omicron variants to rabbit and hare ACE2s reveals the expanding host tendency**

Kaiyuan Shi,^a,b^ Linjie Li,^b^ Chunliang Luo,^b,c^ Zepeng Xu,^b,d^ Baihan Huang,^b,d^Sufang Ma,^b^ Kefang Liu,^b^ Guanghui Yu,^a,#^ George F. Gao^b,#^

^a^ Hubei Provincial Key Laboratory for Protection and Application of Special Plants in Wuling Area of China, College of Life Sciences, South-Central Minzu University, Wuhan, China.

^b^ CAS Key Laboratory of Pathogen Microbiology and Immunology, Institute of Microbiology, Chinese Academy of Sciences, Beijing, China.

^c^ College of Veterinary Medicine, Shanxi Agricultural University, Jinzhong, China.

^d^ Faculty of Health Sciences, University of Macau, Macau SAR, China.

^#^ Address correspondence to George F. Gao, [gaof@im.ac.cn and Guanghui Yu, yusheen@163.com](mailto:%20wangqihui@im.ac.cn).

Kaiyuan Shi and Linjie Li contributed equally to this work.

**Supplementary Figures**

**
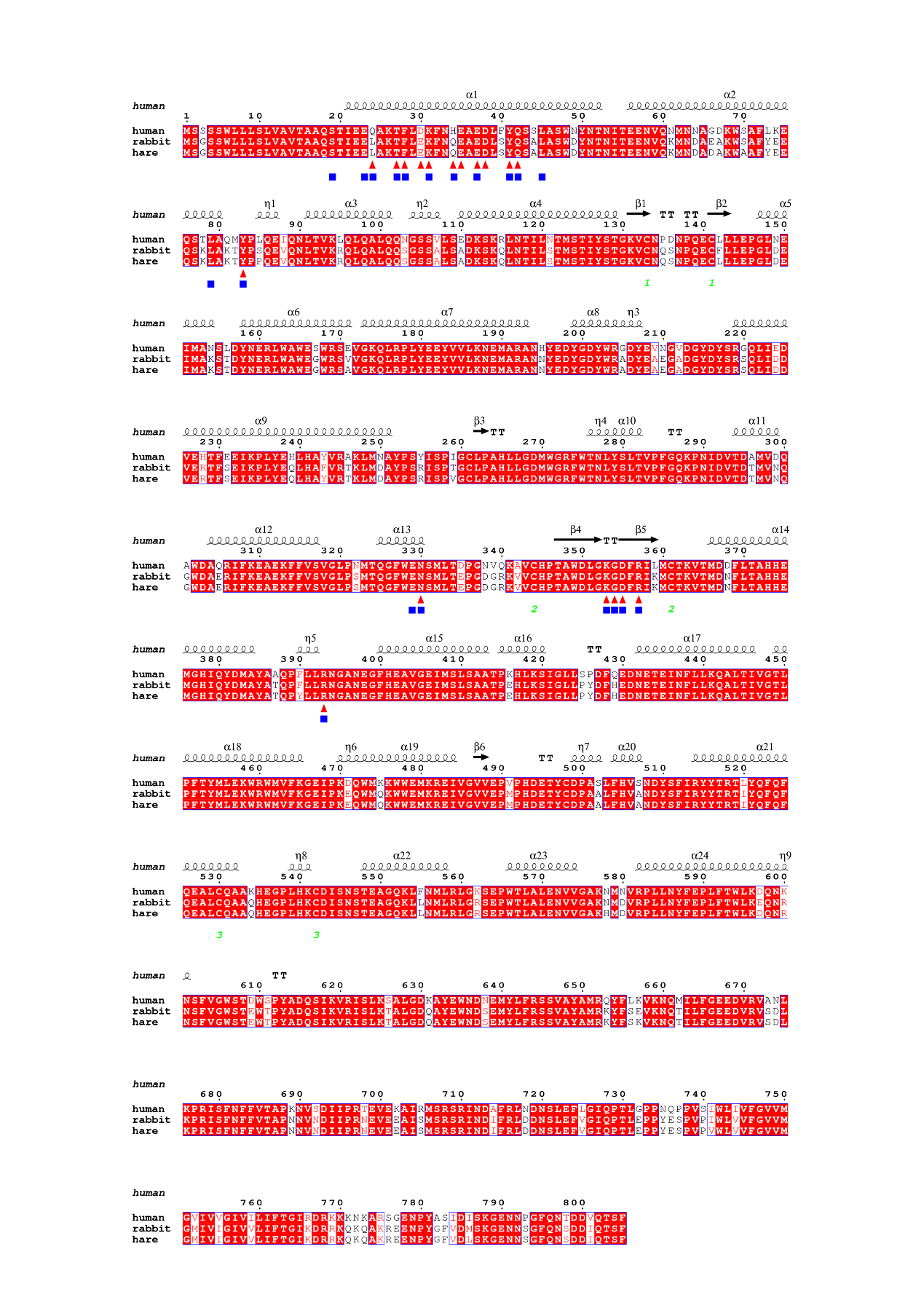
**

**Figure S1 Structure based sequence alignment of human, rabbit and hare ACE2**

Residues contacting the PT RBD and SARS-CoV RBD of rabbit ACE2 are labeled with red triangles and blue squares, respectively. Identical residues are highlighted in white on a red background, and residues in red on a white background indicate a similarity score >0.7, considering physio-chemical properties. The alignment was performed by using T-COFFEE and visualized by ESPript 3.0.


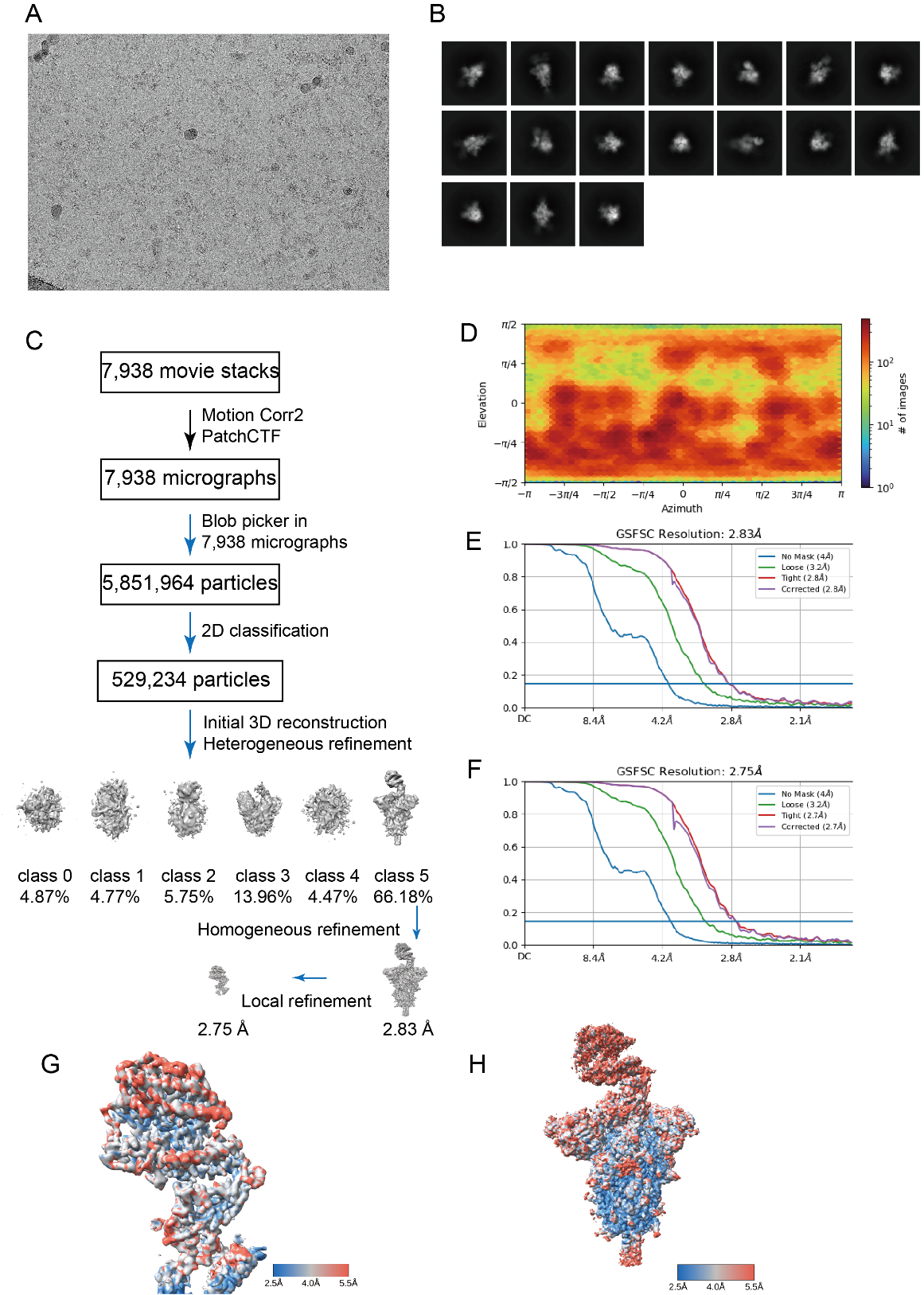


**Figure S2 Cryo-EM data processing of PT S/rabbit ACE2 complex**

(A) A representative electron micrograph. (B) Selected 2D class averages of the SARS-CoV-2 PT S/rabbit ACE2 complex. (C) Schematic illustration of data processing steps. (D) Angular distribution of the particles. (E) Resolution estimation of the global volume. (F) Resolution estimation of the local-refined volume. (G, H) Resolution distribution of the global volume (G) and the local-refined volume (H), where blue represents for high resolution area, and red represents for low resolution area.


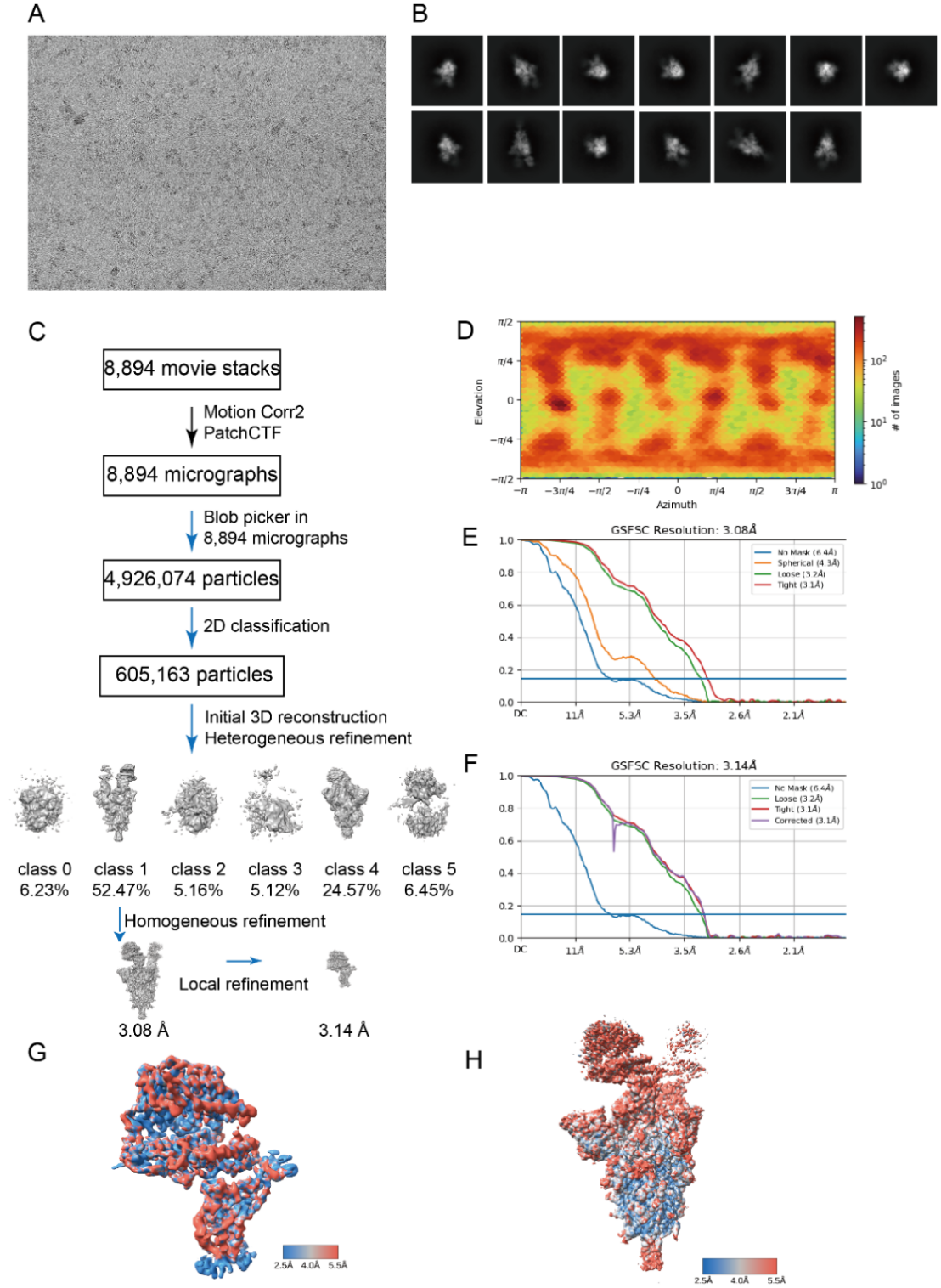


**Figure S3 Cryo-EM data processing of Omicron BA.4/5 S/rabbit ACE2 complex**

(A) A representative electron micrograph. (B) Selected 2D class averages of the Omicron BA.4/5 S/rabbit ACE2 complex. (C) Schematic illustration of data processing steps. (D) Angular distribution of the particles. (E) Resolution estimation of the global volume. (F) Resolution estimation of the local-refined volume. (G, H) Resolution distribution of the global volume (G) and the local-refined volume (H), where blue represents for high resolution area, and red represents for low resolution area.


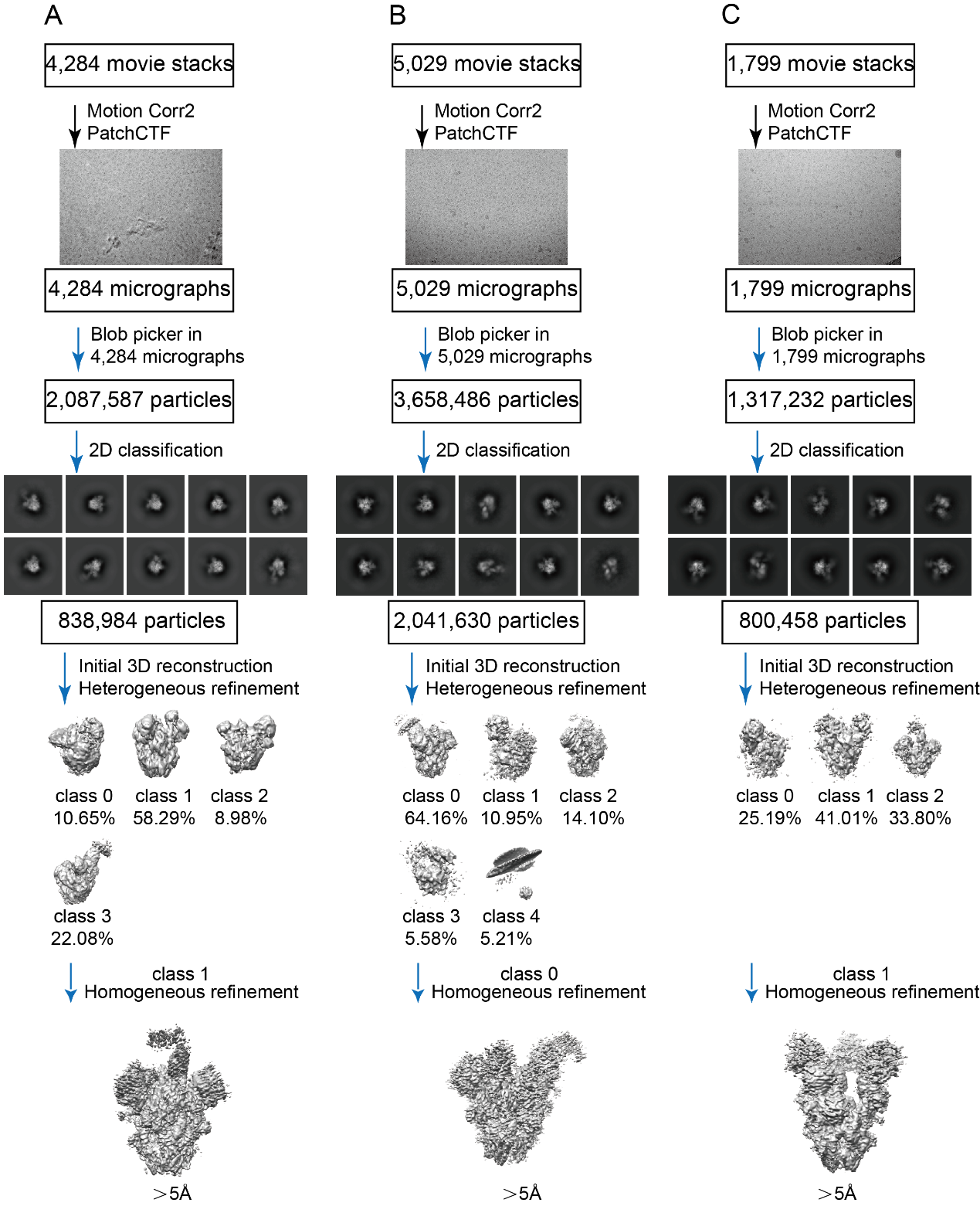


**Figure S4 Cryo-EM data processing of SARS-CoV S/rabbit ACE2 complex**

Data processing workflow for SARS-CoV S/rabbit ACE2 complex when the molar ratio of S/ACE2 is 1:1 (A), 1:3 (B) and 1:5 (C)


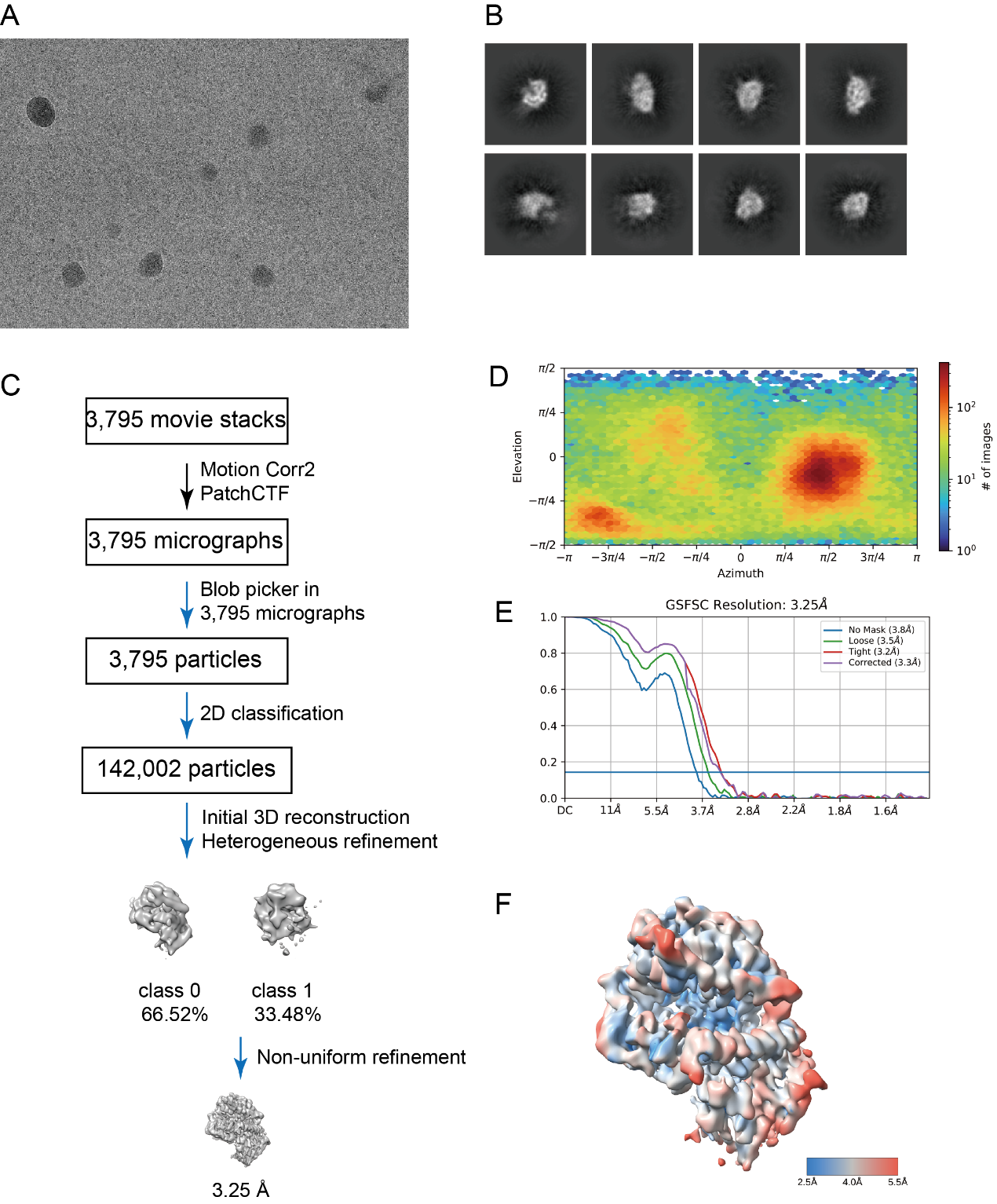


**Figure S5 Cryo-EM data processing of SARS-CoV RBD/rabbit ACE2 complex**

(A) A representative electron micrograph. (B) Selected 2D class averages of the SARS-CoV RBD/rabbit ACE2 complex. (C) Schematic illustration of data processing steps. (D) Angular distribution of the particles. (E) Resolution estimation of the final volume. (F) Resolution distribution of the final volume, where blue represents for high resolution area, and red represents for low resolution area.

**Supplementary Table**

**Table S1 Cryo-EM data collection, processing and refinement statistics.**

|  | PT S/rabbit ACE2 | BA.4/5 S/rabbit ACE2 | SARS-CoV RBD/rabbit ACE2 |
| --- | --- | --- | --- |
| Data collection and processing | | |  |
| Microscope | Titan Krios G3i | Titan Krios G3i | Titan Krios G3i |
| Magnification | 82k | 82k | 105k |
| Voltage (kV) | 300 | 300 | 300 |
| Electron exposure (e^-^/Å^2^) | 50 | 50 | 60 |
| Defocus range (μm) | -1.0 ~ -2.0 | -1.0 ~ -2.0 | -1.0 ~ -2.0 |
| Pixel size (Å) | 0.88 | 0.88 | 0.69 |
| Symmetry imposed | C1 | C1 | C1 |
| Final particle images (no.) | 22539 | 300833 | 94455 |
| Map resolution (Å) | 2.83Å/2.75Å | 3.08Å/3.14Å | 3.25 Å |
| FSC threshold | 0.143 | 0.143 | 0.143 |
| Refinement |  |  |  |
| Initial model used  (PDB code) | 7DF4 | 7DF4 | 2AJF |
| Non-hydrogen atoms | 6348 | 6354 | 6244 |
| Protein residues | 782 | 780 | 768 |
| Validation |  |  |  |
| Clash score | 8.71 | 8.54 | 9.92 |
| Poor rotamers | 1.32 | 0.29 | 0.15 |
| R.m.s. deviations |  |  |  |
| Bond length (Å) | 0.004 | 0.004 | 0.003 |
| Bond angles (°) | 0.722 | 0.870 | 0.704 |
| Ramachandran statistics (%) |  |  |  |
| Most favored | 95.49 | 95.49 | 94.24 |
| Allowed | 4.51 | 4.51 | 5.76 |
| Disallowed | 0.00 | 0.00 | 0.00 |
